# Resveratrol Promotes Hypertrophy in Wildtype Skeletal Muscle and Reduces Muscle Necrosis and Gene Expression of Inflammatory Markers in *Mdx* Mice

**DOI:** 10.3390/molecules26040853

**Published:** 2021-02-06

**Authors:** Keryn G. Woodman, Chantal A. Coles, Shireen R. Lamandé, Jason D. White

**Affiliations:** 1 Murdoch Children’s Research Institute, Royal Children’s Hospital, Parkville, VIC 3052, Australia; Shireen.lamande@mcri.edu.au; 2 Faculty of Veterinary and Agricultural Science, The University of Melbourne, Parkville, VIC 3010, Australia; jwhite@csu.edu.au; 3 Department of Genetics, Yale University, New Haven, CT 06510, USA; 4 Department of Paediatrics, The University of Melbourne, Parkville, VIC 3010, Australia; 5 Office of the Pro Vice Chancellor Research and Innovation, Charles Sturt University, Wagga, NSW 2678, Australia

**Keywords:** muscle, muscular dystrophy, mdx, resveratrol, nutraceuticals, duchenne, inflammation

## Abstract

Duchenne muscular dystrophy (DMD) is a progressive fatal neuromuscular disorder with no cure. Therapies to restore dystrophin deficiency have been approved in some jurisdictions but long-term effectiveness is yet to be established. There is a need to develop alternative strategies to treat DMD. Resveratrol is a nutraceutical with anti-inflammatory properties. Previous studies have shown high doses (100–400 mg/kg bodyweight/day) benefit *mdx* mice. We treated 4-week-old *mdx* and wildtype mice with a lower dose of resveratrol (5 mg/kg bodyweight/day) for 15 weeks. Voluntary exercise was used to test if a lower dosage than previously tested could reduce exercise-induced damage where a greater inflammatory infiltrate is present. We found resveratrol promoted skeletal muscle hypertrophy in wildtype mice. In dystrophic muscle, resveratrol reduced exercise-induced muscle necrosis. Gene expression of immune cell markers, CD86 and CD163 were reduced; however, signalling targets associated with resveratrol’s mechanism of action including Sirt1 and NF-κB were unchanged. In conclusion, a lower dose of resveratrol compared to the dosage used by other studies reduced necrosis and gene expression of inflammatory cell markers in dystrophic muscle suggesting it as a therapeutic candidate for treating DMD.

## 1. Introduction

Duchenne muscular dystrophy (DMD) is a progressive neuromuscular disorder, that arises from mutations in the dystrophin gene [1] and leads to the absence or severe deficiency of the dystrophin protein [2]. Corticosteroids are used in DMD patients to prolong ambulation and maintain respiratory health; however, they have adverse side effects [3], [4]. Many types of therapeutics are in development for DMD and include gene correction strategies, exon skipping and utrophin upregulation; some of these therapies are in the initial stages of clinical use [5]. Despite these advances, there is still a need to develop alternative strategies to treat DMD which could be implemented in the short term, especially for patients with mutations not amenable to current trials; or in the longer term as an adjunct to corticosteroids and gene correction strategies.

Nutraceutical use is increasingly popular and this trend has not escaped the DMD community [6]. Whilst nutraceuticals are not able to correct the genetic defect in DMD, many have anti-inflammatory or antioxidant properties which could help dampen the chronic inflammation in DMD patients alongside corticosteroids. Some nutraceuticals with antioxidant or anti-inflammatory properties that have been trialled clinically in DMD include co-enzyme Q10 and green tea extract (reviewed in [6]). Whilst these trials were small in terms of the number of patients the co-enzyme Q10 study showed some encouraging results in terms of muscle performance measured by quantitative muscle testing (QMT). Other pharmacological drugs such as vamorolone have been shown to reduce inflammation and cardiomyopathy in DMD mouse models [7]. Given that many nutraceuticals have documented safety profiles, are already routinely used for other health issues and are available over the counter, they can be readily implemented into a clinical setting for DMD.

Resveratrol (3,5,4-trihydroxy-trans-stilbene), a phytoalexin found in numerous plant species, exerts its beneficial effects on many pathways and targets such as glucose homeostasis, lipid metabolism, insulin sensitivity, reactive oxygen species (ROS), carcinogenicity, inflammation, mitochondrial biogenesis, cell cycle regulation and apoptosis [6,8,9]. Many of resveratrol’s actions are due to the activation of sirtuin 1 (Sirt1). Resveratrol allosterically binds to the N terminus of Sirt1 thus activating it [10]. Sirt1 subsequently deacetylates a range of downstream signalling targets in a variety of tissue types.

A series of studies have considered the effect of resveratrol on the myogenic C2C12 cell line [11,12,13]. Resveratrol increased myoblast elongation and differentiation and this was associated with increased expression of the myogenic regulatory factors (MRFs) *Myod* and *Myog* [11]. In another study, resveratrol increased the expression of *Myf5* and *Myod* during the proliferation phase and *Myog* and *Myhc* during the differentiation phase [12]. Studies using the Sirt1 inhibitor, nicotinamide, directly link the effect of resveratrol on C2C12 cells with Sirt1 expression; proliferation was decreased with nicotinamide treatment [13].

The in vitro effects of resveratrol on myogenesis led to a series of studies in the *mdx* mouse, which is a widely used mouse model of human DMD. The studies used different resveratrol doses at varying ages and duration of treatment. Delivery of 100mg/kg/day for eight weeks resulted in a significant reduction in bodyweight and extensor digitorum longus (EDL), soleus and tibialis anterior (TA) muscle weights, oxidative damage and fibrotic tissue in 41-week old mice [14]. In the same study, fatigue resistance was increased by approximately 20% in the soleus muscle and the EDL and soleus muscles were protected from contraction-induced injury. A high mortality rate is seen with high doses of resveratrol (400 mg/kg/day) [14]. Another study delivered resveratrol to *mdx* mice via oral gavage for 10 days at 10, 100 or 500 mg/kg/day; only the 100 mg/kg dose significantly increased *Sirt1* gene expression and thus was the only dose considered in subsequent analysis [15]. This was associated with decreased immune cell infiltration in the gastrocnemius muscle but no reduction in gene expression of the pro-inflammatory cytokine *TNF-a*. In a later study 100 mg/kg resveratrol treatment did not improve grip strength or fatigue in the *mdx* mice but produced a significant improvement in rotarod performance and in situ peak tension [16].

Overall, the effect of resveratrol administration in the *mdx* mouse investigated in these four studies shows resveratrol improves muscle function, decreases inflammatory infiltrate and reduces fibrotic tissue. However, there are some inconsistencies between the studies. Whilst all of the studies showed improvements in dystrophic pathology; only one study showed reduced inflammatory infiltrate [16]. This could be due to sampling after resveratrol treatment occurring after peak inflammation timing in the *mdx* mice [14,17]. Considering improvements in muscle function and muscle pathology seen in these studies, we wanted to investigate if inducing greater damage in *mdx* mice would more rigorously test resveratrol’s anti-inflammatory properties when using a lower dose of 5 mg/kg/day. We have previously shown voluntary exercise induces greater damage in *mdx* mouse muscle and thus is more representative of the human pathology [18,19]. In this study, we treated *mdx* mice with a lower dose of resveratrol (5 mg/kg bodyweight/day) than previously tested [14,15,16,17] and used voluntary exercise to induce more dystrophic damage to better assess resveratrol’s anti-inflammatory properties.

## 2. Results

### 2.1. Resveratrol Increased Hypertrophy in Wildtype Mice

**Figure 1:**
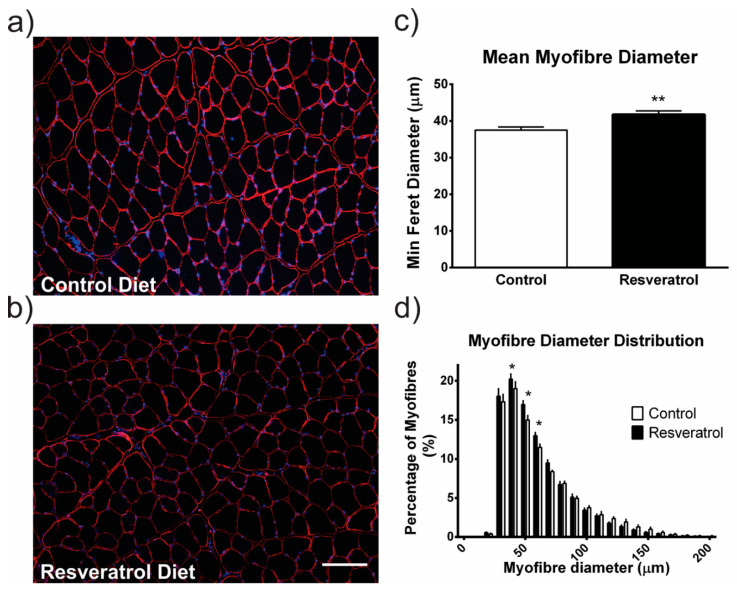
Frozen quadriceps sections were stained with laminin α2 (red) which outlines the individual myofibres and Hoechst (blue) which stains the nuclei. (**a**) Representative image of a stained quadriceps from a mouse on the control diet. (**b**) Representative image of a resveratrol treated mouse quadriceps. (**c**) The average myofibre diameter within the quadriceps muscle is significantly larger in the resveratrol treated cohort in comparison to the control cohort. (**d**) The myofibres were grouped into 10 μm intervals and the proportion of myofibres in each interval was plotted in a histogram. There were more myofibres measuring between 50 and 70 μm in the resveratrol treated group in comparison to the control group. The white scale bar indicates 200 μm. Graphs show mean ± SEM. * indicates *p* < 0.05, ** indicates *p* < 0.001. n = 5 for each treatment group.

We first investigated the effect of the resveratrol diet on healthy muscle. The average myofibre diameter was larger in the resveratrol treated wildtype mice compared to the mice on the control diet (*p* < 0.001) (Figure 1a–c). In resveratrol treated wildtype mice there was a shift to larger myofibre diameters with more myofibres ranging from 50 to 70 μm in diameter when compared to the wildtype mice on the control diet (*p* < 0.05) (Figure 1d).

### 2.2. Exercise-Induced Necrosis is Reduced in Mdx Mice with Resveratrol Treatment

**Figure 2:**
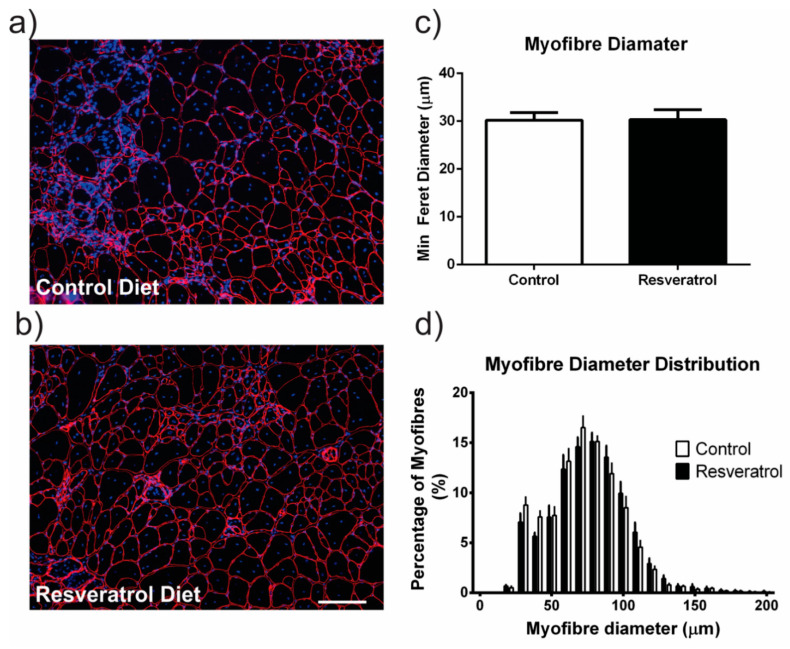
Frozen *mdx* quadriceps sections were stained with laminin α2 (red) and Hoechst (blue). (**a**) Representative image of a stained quadriceps from an *mdx* mouse on the control diet. (**b**) Representative image of a resveratrol treated *mdx* mouse quadriceps. (**c**) The average myofibre diameter within the *mdx* quadriceps muscle is unchanged with resveratrol treatment when compared to the controls. (**d**) The myofibres were grouped into 10 μm intervals and the proportion of myofibres in each interval was plotted in a histogram. There was no change in myofibre distribution with resveratrol treatment. The white scale bar indicates 200 μm. Graphs show mean ± SEM. n = 5 for each treatment group.

**Figure 3:**
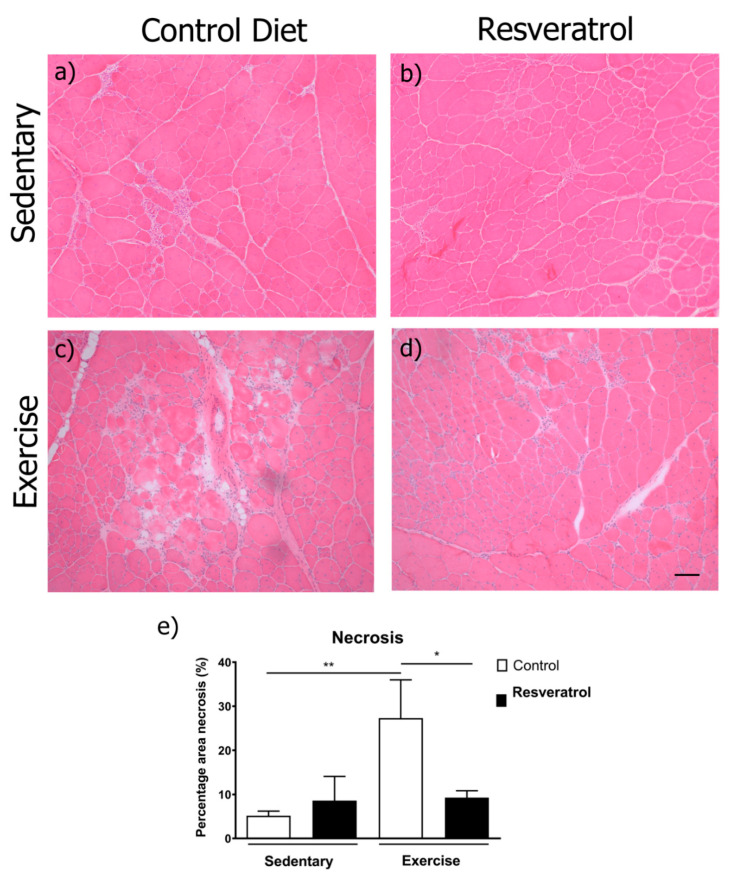
Transverse quadriceps sections were stained with haemotoxylin and eosin to illustrate muscle architecture, areas of inflammation and fibrosis. (**a**) Representative image of an *mdx* control diet sedentary quadriceps, (**c**) an *mdx* control diet exercise quadriceps, (**b**) a resveratrol treated sedentary *mdx* quadriceps and (**d**) an *mdx* resveratrol treated exercised quadriceps. (**e**) Areas of necrosis were quantified and expressed as a percentage of the total quadriceps area. Necrosis was not different with resveratrol treatment in the sedentary *mdx*. Voluntary exercise significantly increased necrosis. This effect is not observed in the resveratrol treated mice when comparing sedentary with exercised resveratrol treated mice. The black scale bars represent 100 μm. Graphs show mean ± SEM. * indicates *p* < 0.05, ** *p* < 0.01. n = 5 for each treatment group.

Despite an increase in myofibre diameter in wildtype mice, resveratrol did not change the average myofibre diameter or the myofibre size distribution in *mdx* mice (Figure 2a–d). We next examined the muscle for pathological features. Necrosis is a measure of damage in dystrophic muscle and includes areas of mononuclear cells predominantly representing inflammatory infiltrate, myofibres with fragmented sarcoplasm and areas of regenerating myofibres. To determine if resveratrol administration reduced skeletal muscle necrosis in the *mdx* quadriceps muscle, the frozen transverse sections were stained with haematoxylin and eosin (representative images in Figure 3a–d) and necrotic areas were quantitated. The resveratrol treatment did not alter necrosis in the quadriceps of the sedentary *mdx* mice when compared to the control diet cohort (Figure 3e). As in previous studies [18,19], voluntary exercise increased necrosis and damage in *mdx* mice (Figure 3e). Resveratrol treatment protected the muscle from this increase in exercise-induced damage (Figure 3e).

### 2.3. Resveratrol Treatment did not Reduce Damaged Myofibres Mdx Mice

**Figure 4:**
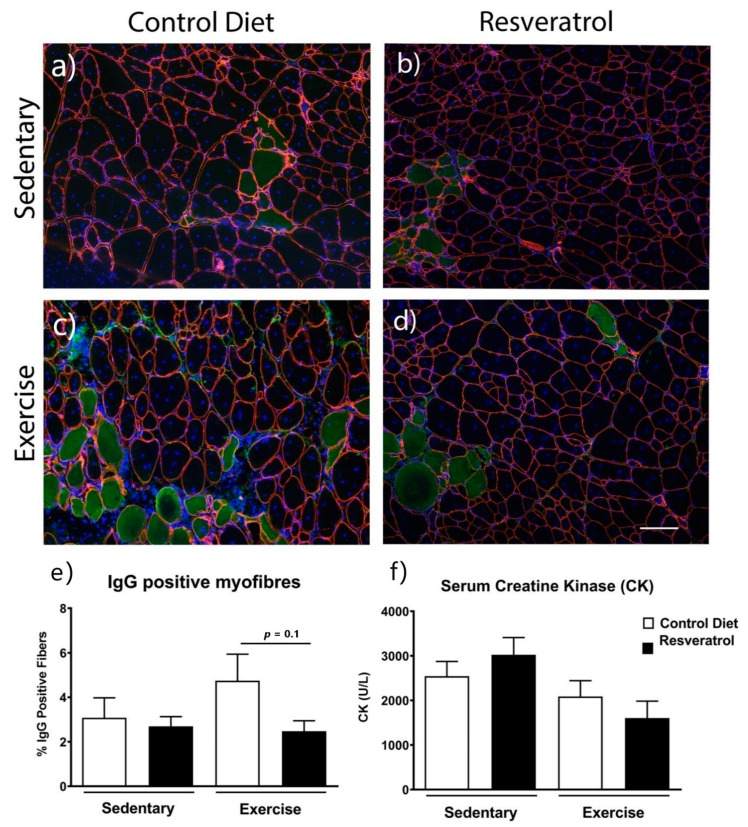
Quadriceps muscles were stained with laminin α2 (red), nuclei with Hoechst (blue) and IgG (green) which enters fibres with impaired sarcolemma. Representative image of (**a**) an *mdx* control diet sedentary quadriceps, (**c**) a control diet exercise *mdx* quadriceps, (**b**) a resveratrol treated sedentary *mdx* quadriceps and a (**d**) resveratrol treated exercise *mdx* quadriceps. (**e**) Quantitative assessment of the quadriceps muscle shows the percentage of damaged IgG positive fibres is unchanged with resveratrol treatment in both exercise and sedentary groups. (**f**) Serum CK activity was similar in all cohorts. The white scale bars represent 200 μm. Graphs show mean ± SEM. n = 5 for each treatment group.

To determine if resveratrol treatment reduced damaged myofibres in the quadriceps of the mdx mice, transverse frozen sections were stained with anti-α2 laminin, Hoechst and anti-IgG (Figure 4a–d). In mdx mice on the control diet, voluntary exercise did not increase the percentage of myofibres positive for anti-IgG (Figure 4e). There was no difference in the percentage of myofibres staining positive for anti-IgG with the resveratrol treatment in either the sedentary or exercised groups compared with mdx on the control diet (Figure 4e). Serum CK activity was also not reduced with resveratrol treatment in either the sedentary or exercise groups when compared to the respective control diet cohorts (Figure 4f).

### 2.4. Resveratrol Administration Decreases Gene Expression of Immune Cell Markers

**Figure 5:**
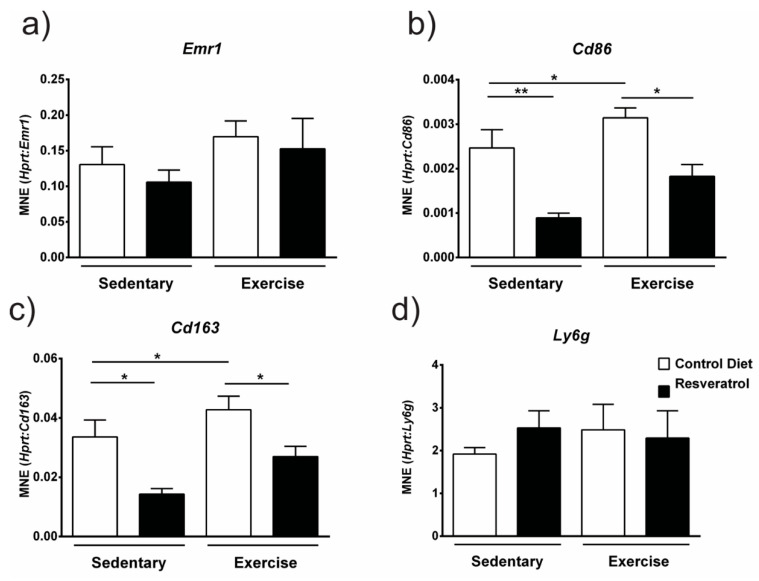
(**a**) Expression of *Emr1* a marker for (F4/80 positive) macrophages was unchanged with resveratrol treatment. (**b**) Expression of *Cd86*, a marker for M1 macrophages and (**c**) Cd163, a marker for M2 macrophages was downregulated with resveratrol treatment in sedentary *mdx*. Voluntary exercise resulted in increased expression of M1 (**b**) (*Cd86*) and M2 (**c**) (*Cd163*) gene expression. Resveratrol treatment was effective in preventing this increase in exercised *mdx*. (**d**) Expression of the neutrophil marker *Ly6g* was unchanged with resveratrol administration in both sedentary and exercise *mdx*. Graphs show mean ± SEM. * *p* < 0.05, ** *p* < 0.01. n = 5 for each treatment group.

Since necrosis was decreased with resveratrol treatment in exercised mdx muscle we investigated the gene expression of inflammatory cell markers. Expression of the pan-macrophage marker F4/80 (Emr1) was not changed by resveratrol treatment in either the sedentary or exercised mdx quadriceps (Figure 5a). However, when we assessed Cd86, a cell surface marker for cytotoxic M1 macrophages (Figure 5b) and Cd163 a marker for M2 macrophages (Figure 5c) we found both were downregulated in resveratrol treated sedentary and exercised mice. In addition, gene expression of both these inflammatory markers was increased with voluntary exercise in the control *mdx* suggesting greater numbers of these cells are involved in the exercise-induced inflammatory infiltrate found in necrotic dystrophic muscle. Neutrophils are also present in necrotic dystrophic muscle; however, we found no difference in expression of the neutrophil marker lymphocyte antigen 6 complex, locus G (*Ly6G*) gene with resveratrol treatment in either sedentary or exercise groups (Figure 5d). These data suggest resveratrol exerts its anti-inflammatory effects in dystrophic muscle on macrophage sub-types that are predominant during muscle repair [20].

### 2.5. Resveratrol Treatment Increased Gene Expression of Il6 and Tnf

**Figure 6:**
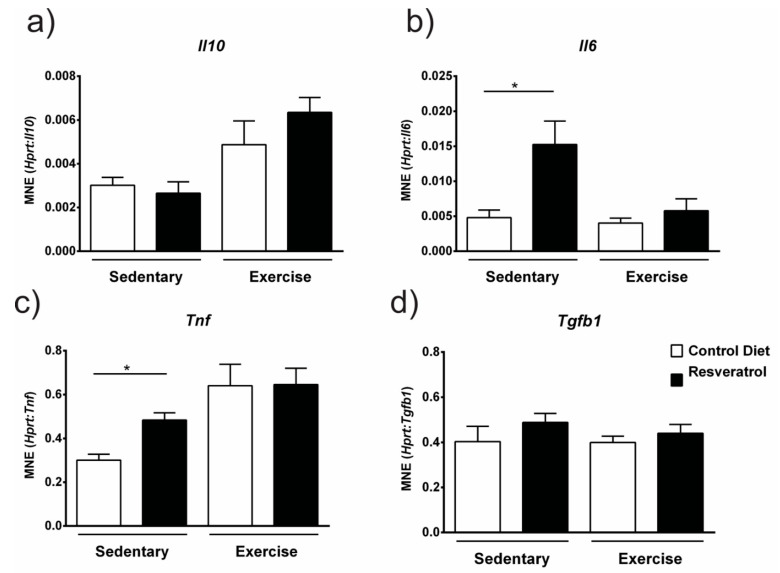
(**a**) *Il-10* gene expression is unchanged with resveratrol treatment. (**b**) *Il-6* gene expression was up-regulated with resveratrol treatment in the sedentary *mdx* mice but remained unchanged in the exercised group. (**c**) Gene expression of *TNF-α* was up-regulated with resveratrol treatment in the sedentary *mdx* mice, yet remained unchanged in the exercised group. (**d**) Gene expression of *Tgf-β* was unchanged with resveratrol treatment. There was also no effect of exercise on gene expression of these pro-inflammatory cytokines. Graphs show mean ± SEM. * *p* < 0.05. n = 5 for each treatment group.

As gene expression of an M1 macrophage marker Cd86 was downregulated with resveratrol treatment, we assessed if this was accompanied by a decrease in inflammatory cytokine expression. Gene expression of interleukin 10 (*Il-10*) remained unchanged with resveratrol administration (Figure 6a) in both sedentary and exercised *mdx*. Interestingly, expression of two cytokines, interleukin 6 (*Il6*) (Figure 6b) and tumour necrosis factor (*Tnf*) (Figure 6c) were significantly increased with resveratrol treatment in the sedentary mice (*p* < 0.05) yet remained unchanged in the exercised mice (Figure 6b–c). Resveratrol treatment did not change the expression of transforming growth factor-beta (*TGF-β*) in either the sedentary or control groups when compared to the controls (Figure 6d).

### 2.6. Resveratrol Treatment does not Alter Sirt1 or Nfb gene Expression in Mdx Mice

**Figure 7:**
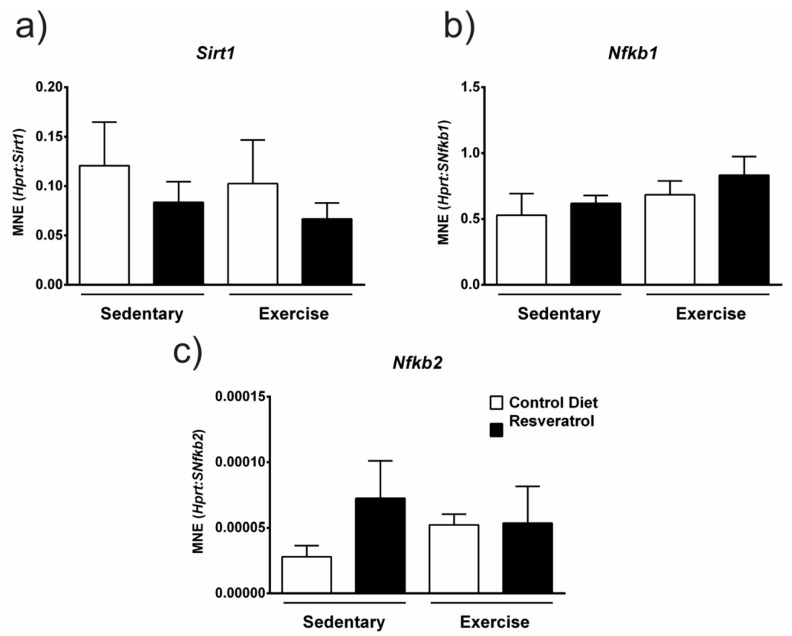
(**a**) *Sirt1* gene expression is unchanged with resveratrol treatment in both sedentary and exercise groups when compared to respective controls. (**b**) Gene expression of *NF-κB1* is unchanged with resveratrol treatment in both the sedentary and exercise cohorts. (**c**) Likewise, *NF-κB2* gene expression is not significantly altered with resveratrol administration in either sedentary or exercised *mdx* mice. Graphs show mean ± SEM. n = 5 per treatment group.

Resveratrol is thought to elicit its anti-inflammatory and antioxidant effects through downstream signalling targets such as Sirt1 and NF-κB. Sirt1 belongs to the NAD+-dependent protein deacetylase family or sirtuins, which have key roles in many cellular processes including inflammation and oxidative stress. Resveratrol can regulate Sirt1 expression [21,22]. The NF-κβ pathway is another target of resveratrol and has critical roles in inflammation, immunity, cell proliferation, differentiation and survival [23]. We assessed if resveratrol altered gene expression of these signalling targets. Sirt1 (Figure 7a), *Nfkb1* (Figure 7b) or *Nfb2* (Figure 7c) gene expression was unchanged with resveratrol administration in both the sedentary and exercise mdx.

## 3. Discussion

Whilst previous studies have shown that treating *mdx* mice with resveratrol has beneficial effects on muscle pathology and performance, only one study showed a positive effect on immune infiltration [14,15,16,17]. This inconsistency could be explained by the fact that the *mdx* mouse has a milder disease pathology than the human condition with peak inflammation occurring between 6 and 12 weeks of age [24]. The age at which muscles were harvested for immune cell analysis and therefore the amount of immune infiltration could be a major factor in determining resveratrol’s ability to suppress inflammation. When tissues were harvested around the time of peak inflammation, resveratrol treated samples had fewer muscle leukocytes (CD45+ cells) and F4/80 positive macrophages [15]. By contrast, when the tissue was harvested well beyond the period of peak inflammation no differences in immune infiltration were seen in the resveratrol treated mice [16]. Another group also found no change in total immune infiltration (CD45+ cells) after treating *mdx* mice with resveratrol for 32 weeks from 9 weeks of age [17]. Based on this we used voluntary exercise to increase muscle damage and necrosis and thus immune infiltration in mdx and tested if administration of resveratrol at a much lower dose would protect against exercise-induced damage. We found where there is greater necrotic tissue in *mdx* mice due to exercise-induced damage, resveratrol is able to protect the dystrophic muscle from contraction-induced injury at a much lower dose (5 mg/kg bodyweight/day) than previously shown to be beneficial (100–400 mg/kg bodyweight/day). This suggests there needs to be a certain amount of inflammation present in the muscle tissue for the effects of resveratrol to be clearly seen and thus timing in the *mdx* mouse is critical to ensure this.

In necrotic *mdx* tissue, the immune infiltrate is comprised of macrophages, T-cells, neutrophils, eosinophils and mast cells; however, the number of macrophages greatly outnumbers the other immune cells [25]. Due to resveratrol’s effects on reducing exercise-induced necrosis in this study, we investigated the gene expression of immune cell markers expressed by macrophages and neutrophils. There are two distinct populations of macrophages; the M1 “cytotoxic” population release pro-inflammatory cytokines, and can promote cell lysis and phagocytosis of necrotic myofibres, clearing the way for muscle regeneration [26], whilst the M2 anti-inflammatory macrophages release anti-inflammatory cytokines and factors which attenuate tissue repair, such as IL-10 and TGF-β [27]. In *mdx* mice; however, the normal inflammation and regeneration process is disrupted and the normally well-coordinated inflammatory process and muscle repair is impaired and M1 macrophages continue to be present in the muscle. The shift from M1 to M2 macrophages is instead replaced with co-invasion of M2 macrophages with M1 macrophages and neutrophils [28]. The M2 macrophages produce IL-10 in an immunomodulation attempt to deactivate the M1 macrophages. IL-10 and IL-4 are able to modulate macrophage polarisation from the M1 to M2 phenotype and thus reduce inflammation and damage in *mdx* mice [28]. We show that exercise increased the gene expression of *Cd86*, a marker of cytotoxic M1 macrophages and *Cd163*, a marker of anti-inflammatory M2 macrophages. This increase was dampened with resveratrol treatment. The gene expression of these inflammatory markers was also decreased in sedentary *mdx* with resveratrol treatment suggesting these macrophage subsets are directly impacted by resveratrol irrespective of the magnitude of an inflammatory infiltrate. In a murine myocardial infarction model, resveratrol treatment reduced damage and fibrosis and that resveratrol was cardioprotective by suppressing inflammation [29]. They also showed via flow cytometry that CD11c+ M1 macrophages were significantly decreased with resveratrol treatment [29]. Overall, these data highlight resveratrol’s anti-inflammatory role and suggests that it could potentially have a role in macrophage polarisation during muscle injury.

Another recent study investigated resveratrol’s actions on human THP-1 monocytes and macrophages in vitro [30]. They first showed that the addition of resveratrol at a concentration of 5 µmol/L inhibited proliferation of THP-1 monocytes and at >10 µmol/L concentrations resveratrol induced cell apoptosis and caused G0/G1 phase arrest in vitro [30]. The reduced necrosis and decreased gene expression of M1 and M2 inflammatory macrophages found in our treated exercised *mdx* mice and the reduced immune cell infiltration with resveratrol treatment [15] is consistent with this study [30] and suggests that resveratrol has effects on macrophage proliferation/polarisation. Future studies could use flow cytometry to explore the effect of resveratrol on macrophages and other immune cell populations as well as to further elucidate which cell type is responsible for increases in cytokine production such as Il6 and Tnf.

The anti-inflammatory and anti-apoptotic effects of resveratrol are thought to be due to effects on Sirt1 activation and NF-κB inhibition [31,32,33]. Previous research assessed Sirt1 activation with different resveratrol doses and found 100 mg/kg was the most effective in up-regulating Sirt1 mRNA [15]. We, therefore, conclude that our low dose of 5 mg/kg was not sufficient to increase gene expression of *Sirt1* and therefore the results seen in our cohort are likely attributed to effects on inflammation and are therefore not mediated by Sirt1. This effect was also observed for *NF-κB1* and *NF-κB2* which were unchanged by resveratrol treatment in the *mdx* mice again suggesting that resveratrol’s effects in *mdx* mice are due to its ability to suppress inflammation, perhaps through its effects on other signalling targets such as the p38 MAPK pathways [34]. Overall resveratrol has shown potential to reduce pathology in the *mdx* mouse model of DMD and highlights the need for this to be tested clinically. A recent 2020 study tested resveratrol supplementation in an open-label, single-arm, phase IIa trial in 11 patients with Duchenne, Becker or Fukuyama muscular dystrophies [35]. Whilst this study was quite small, they were able to show a significant reduction in creatine kinase levels and improved motor function measurements [35]. These results are highly encouraging and open the door for trials with increased patient numbers.

Whilst we have shown beneficial effects in *mdx* mice, we also investigated resveratrol’s ability to affect healthy muscle. Previous in vitro studies demonstrate that resveratrol increases myoblast differentiation in C2C12 cells and in primary myoblasts [11,12], [36]. Here we show a significant increase in the average myofibre diameter, particularly in fibres which are larger than average, in the quadriceps muscle of resveratrol treated wildtype mice. These findings correlate with a study that found increased myofibre hypertrophy in resveratrol treated mice (25 mg/kg for 4 weeks) that underwent a repetitive ladder-climbing protocol [37]. Whilst changes in muscle mass and metabolic markers were seen, the underlying mechanism behind the increased hypertrophy was not explored [37]. Together these data suggest that resveratrol treatment promotes muscle hypertrophy in wildtype mice and this could have direct applications to the livestock industry, sports medicine or potentially for treating cachexia.

## 4. Materials and Methods

### 4.1. Mice and Trial Design

All animal experiments were approved by the University of Melbourne Animal Ethics Committee (AEC) and the Murdoch Children’s Research Institute AEC. Male C57BL10/ScSn (wildtype) and C57Bl10/mdx (*mdx*) mice were obtained from Animal Resources Centre (Perth, WA, Australia). At 4 weeks of age (after weaning and prior to the peak period of inflammation in *mdx* mice [24]), 12 mice received either a control diet or resveratrol diet (5 mg/kg bodyweight/day) for 12 weeks. After 12 weeks the groups were divided into sedentary and exercised groups. Mice were housed singly with running wheels for three weeks, with wheel rotation data being recorded as described [18]. The trial design included treatment of sedentary mice for 12 weeks followed by 3 weeks of further treatment with voluntary exercise. Peak inflammation occurs at 6–12 weeks of age [24] and is exacerbated by adding the voluntary exercise protocol. The trial design thus ensured robust testing of resveratrol’s anti-inflammatory properties. The mice continued to receive their specified diet over the exercise period. The quadriceps were chosen for further analyses as they are highly susceptible to exercise-induced damage [18]. At trial completion blood was obtained via cardiac puncture and the quadriceps muscles were harvested and snap-frozen. The contralateral quadriceps muscles from the remaining hindlimb were mounted in 5% tragacanth (*w/v*) (Sigma, St Louis, MO, USA) and frozen in liquid nitrogen-cooled isopentane (Sigma, St Louis, MO, USA) and stored at −80 °C for histological analyses.

### 4.2. Immunostaining

The quadriceps muscle was cryosectioned (10 μm) and blocked in 10% (*v/v*) donkey serum (Millipore, Billerica, MA, USA) in wash buffer (0.1% Tween, 0.5% Bovine albumin serum in PBS) before immunodetection of rat anti-laminin α-2 (Santa Cruz Biotechnology, Dallas, TX, USA) to identify myofibre boundary. Sections were imaged on a Zeiss Axio Imager M1 upright fluorescent microscope with an AxioCam MRm camera running AxioVision software V4.8.2.0 (Carl Zeiss, Oberkochen, Germany, 2015).

### 4.3. Histology and Morphometric Analysis

#### 4.3.1. Minimum Feret’s Diameter

Image J version 1.48G (U. S. National Institutes of Health, Bethesda, MD, USA) was used to calculate minimum Feret’s diameter as described in Treat-NMD standard operating procedure “Quantitative determination of muscle fibre diameter” (http://www.treat-nmd.eu/downloads/file/sops/dmd/MDX/DMD_M.1.2.001.pdf (accessed on 12 December 2020)).

#### 4.3.2. Damaged Myofibres

To determine the percentage of damaged myofibres, muscles were stained for intracellular IgG using AlexaFluor donkey anti-mouse IgG 488 (Thermofisher Scientific, Waltham, MA, USA), Anti-IgG positive myofibres in the entire muscle cross-section were counted manually and expressed as a percentage of the total myofibre number.

#### 4.3.3. Central Nuclei and Necrosis

Quadriceps were cryosectioned (10 µm) and stained with hematoxylin and eosin. The necrotic area and percentage of central nuclei were calculated for entire muscle cross-sections as previously described [18].

#### 4.3.4. Creatine Kinase Enzyme Activity

Blood was centrifuged at 12,000 g for 15 min to separate the serum. To measure CK activity, the serum was thawed and 5 μL was aliquoted into a 96 well plate in triplicate, followed by CK-NAC reagent (Thermofisher Scientific, Waltham, MA, USA). The change in absorbance was recorded at 340 nm over three minutes (measured in 20 s intervals) at 37 °C using a Paradigm Detection Platform (Beckman Coulter, Brea, CA, USA) [38].

#### 4.3.5. RNA Extraction, cDNA Synthesis, qPCR and Oligonucleotide Primer Design

RNA was extracted from snap-frozen quadriceps and reverse transcribed to cDNA as previously described [38]. Each qPCR reaction contained 25 ng of cDNA added to “Go Taq Sybr Green” qPCR master mix (Promega, Madison, WI, USA) as per manufacturer’s instructions. All qPCR reactions were performed using a LightCycler480 (Roche Applied Bioscience, Basel, Switzerland).

#### 4.3.6. Statistical Analyses

Where data was normally distributed GraphPad Prism 5 (GraphPad Software, La Jolla, CA, USA) was used to assess statistical significance. Where there was a direct comparison between two groups or two data points, a Student’s t-test was used. RT-qPCR data and data that were not normally distributed, such as myofibre diameter, were assessed using a non-parametric Mann–Whitney U test in GraphPad Prism [39].

## 5. Conclusions

Treating healthy wildtype mice with 5 mg/kg/day of resveratrol increased myofibre diameter and the proportion of larger myofibres. This finding supports in vitro experiments and could have particular relevance to the livestock, sports medicine fields or cachexia fields where increased muscle mass would be beneficial.

Voluntary exercise increased muscle pathology and immune infiltration in the *mdx* mouse and provided a greater therapeutic challenge for the anti-inflammatory resveratrol supplementation. Low dose resveratrol dampened exercise-induced necrosis. Expression of a cytotoxic M1 macrophage marker was reduced as was an M2 macrophage marker, suggesting there were fewer inflammatory cells and highlighting resveratrol’s anti-inflammatory properties. Overall, we show that resveratrol could be beneficial for treating DMD and future experiments could focus on optimising the dose and treatment duration in exercised mice. Combined administration of resveratrol and corticosteroids could have synergistic therapeutic benefits.

## Sample Availability

Samples of the compounds are not available from the authors.
